# Clinical Characteristics and Outcomes of Malignant Glaucoma after Different Procedures Treated with Pars Plana Vitrectomy: A 10-Year Retrospective Study

**DOI:** 10.3390/medicina54040065

**Published:** 2018-09-05

**Authors:** Lina Krėpštė, Reda Žemaitienė, Arūnas Miliauskas

**Affiliations:** Department of Ophthalmology, Medical Academy, Lithuanian University of Health Sciences, 50161 Kaunas, Lithuania; reda.zemaitiene@kaunoklinikos.lt (R.Ž.); arunas.miliauskas@kaunoklinikos.lt (A.M.)

**Keywords:** malignant glaucoma, pars plana vitrectomy, risk factors, complications, intraocular pressure

## Abstract

*Background and objectives:* Despite established common risk factors, malignant glaucoma (MG) remains a rare condition with challenging management. We aimed to analyze differences in risk factors for MG after different surgeries and outcomes after pars plana vitrectomy (PPV). *Materials and Methods:* This retrospective study included cases of MG treated with PPV between January 2005 and December 2015 in the Department of Ophthalmology, Lithuanian University of Health Sciences, Kaunas, Lithuania. *Results:* A total of 39 cases were analyzed: 23 (59%) after cataract surgery, 13 (33.3%) after trabeculectomy, and 3 (7.7%) after other interventions. Characteristics among the groups did not differ. Intraocular lens refractive power was significantly higher in the cataract group, in which intraocular pressure (IOP) before MG was significantly greater in the affected eye. Normotension was achieved in 92.3%, and a normal anterior chamber in 75%. Additional measures included eye drops (*n* = 24), trabeculectomy (*n* = 5), bleb revision (*n* = 2), synechiotomy (*n* = 4), and cyclophotocoagulation (*n* = 1). The proportion of drop-free patients significantly increased after PPV compared with that before MG development (38.5% versus 15.4%). Complications were observed in 11 cases: choroidal detachments with spontaneous resolution (*n* = 2); retinal detachment (*n* = 1); constant mydriasis (*n* = 1), neovascular glaucoma (*n* = 1); obstruction of filtrating zone by iris (*n* = 1) and by blood clot (*n* = 1); posterior synechia formation causing IOP rise (*n* = 4 (all resolved after synechiotomy)). The cataract group experienced significantly fewer complications than the trabeculectomy group (17.4% vs. 53.8%, respectively). *Conclusions:* There were no differences in the risk of MG among the different surgeries. However, higher IOP in the predisposed eye (versus contra-lateral eye) could indicate additional risk of MG after cataract surgery. PPV afforded reliable treatment for MG and the possibility for glaucoma patients to discontinue topical treatment.

## 1. Introduction

Malignant glaucoma (MG) is a condition involving acute shallowing of the anterior chamber (AC), usually accompanied by ocular hypertension, which is not resolved―nor prevented―by an iridotomy. The phrase “malignant glaucoma” was coined by von Graefe, who was the first to describe the condition [1]. Typically, MG occurs after eye surgery (spontaneous cases have also been reported) and may quickly alter the prognosis of otherwise uncomplicated surgery.

Despite established common risk factors, MG remains a relatively rare condition. Although MG varies according to surgery type and study period, the most recent investigation reported an overall incidence of 2% over an 11-year period [2]. The exact pathogenesis of MG remains unclear, although several different mechanisms have been proposed, including entrapment of aqueous humor behind or within the vitreous, accumulation of fluid within the posterior chamber due to direct lens block, pressure difference(s) between the anterior and posterior compartments due to rotation of ciliary processes, or anterior hyaloid face obstruction [3]. Each of these mechanisms may play a role depending on the situation and, perhaps, on the surgery being performed.

We aimed to analyze differences in risk factors among cases of MG after different surgeries, and outcomes after the MG was treated with pars plana vitrectomy (PPV). To our knowledge, this study involved the second largest sample of MGs treated with PPV [4] and the largest group of MGs treated after cataract surgery published to date [5,6].

## 2. Materials and Methods

The present study was performed at the Department of Ophthalmology, Lithuanian University of Health Sciences, a tertiary health care center, located in Kaunas, Lithuania. The study design was approved by Kaunas Regional Research Ethics Committee (P1-BE-2-26/2015). All cases of MG treated with PPV between January 2005 and December 2015 were retrospectively reviewed. Considering the retrospective nature of the study and the use of anonymized patient data, requirements for informed consent were waived.

All cases were treated with complete PPV combined with moderate vitreous trimming, posterior capsulotomy, iridectomy, zonulohyaloidectomy, synechiotomy (if needed) and, in phakic patients, phacoemulsification with intraocular lens (IOL) implantation. Surgeries were performed by four vitreoretinal surgeons.

Operations were started with dissection of the conjunctiva and pars plana incisions (three incisions in phakic eyes and two incisions in pseudophakic eyes). An infusion line was inserted via pars plana incision into the posterior chamber (in phakic eyes) or via a self-sealing clear corneal incision into the anterior chamber (in pseudophakic eyes).

In phakic eyes, the infusion remained turned off and a limited core vitrectomy just to minimize intraocular pressure (IOP) and deepen the AC was performed. Then, standard cataract surgery and IOL implantation followed. After checking the successful penetration of the infusion line, the vitrectomy cutter and the lighter via pars plana incisions were introduced into the posterior chamber, the infusion was turned on, and the residual pars plana vitrectomy was started. A vitrectomy–phacoemulsification–vitrectomy approach in phakic MG cases has been described by Sharma et al. [7].

In pseudophakic eyes, the infusion line was turned on before inserting it into the anterior chamber. Then, the vitrectomy cutter and the lighter via pars plana incisions were introduced into the posterior chamber and the pars plana vitrectomy was started.

First, posterior capsulotomy and peripheral iridotomy with zonulohyaloidectomy with the vitrectomy cutter were performed to create patent posterior–anterior chambers’ communication, then the complete vitrectomy was performed. Pars plana incisions and conjunctiva were closed with 7/0 Vicryl sutures. Intracameral cefuroxime and subconjunctival antibiotic and steroid injections were given at the end of the surgery.

An IOP-lowering and cyclopegic therapy was applied to all cases as adjunctive therapy while waiting for PPV.

Cases were excluded from analysis if the diagnosis of MG, as the cause of ocular hypertension, was doubtful. If both eyes had MG and were treated with PPV, the second eye was included and previous MG status in the other eye was interpreted as a risk factor. Other risk factors or parameters considered were the female sex, age, hyperopia, diagnosis of angle closure glaucoma, axial length, AC depth before surgery, lens thickness, stage of cataract, IOL refractive power in diopters (D), IOP before, during and after MG, and time for MG to develop. PPV outcomes were evaluated according to effective IOP decrease (normotension was considered to be treatment success), deepening of the AC, the need for other measures, and complications.

Statistical analysis was performed using SPSS Version 20 (IBM Corporation, Armonk, NY, USA). The Shapiro-Wilk test was applied to determine how the variables are distributed. If the dataset followed a normal distribution, the mean with standard deviation was calculated, and means between groups were compared using Student’s t-test. For non-normally distributed data, median and interquartile range (IQR) was calculated, and the non-parametric Mann–Whitney U test was used. If data were non-normally distributed and related, the Wilcoxon signed-rank test was chosen. A receiver-operator characteristic curve (ROC) test for identification of cut-off value was used. Qualitative data were compared using the Pearson chi-squared test. The McNemar test was applied on non-normally distributed paired nominal data; *p* ≤ 0.05 was considered to be statistically significant.

## 3. Results

Forty-three MG patients were treated with PPV during the study period, 39 of whom were ultimately included in the final analysis. Twenty-three (59%) cases developed MG after cataract surgery (group 1), 13 (33.3%) after trabeculectomy (group 2 (2 pseudophakic cases)), and 3 (7.7%) after other interventions (group 3). Patients in group 3 had different characteristics: MG development after peripheral iridotomy with neodymium-yttrium-aluminum garnet (Nd: YAG) laser in a 73-year-old woman, who underwent previous cataract surgery, followed by uveitis and trabeculectomy; MG after IOL exchange from the posterior to AC in a 67-year-old man, who underwent previous IOL repair after head trauma-caused IOL dislocation; and MG after covering of corneal transplant with amniotic membrane in a 65-year-old man. MG developed in 1, 20, and 6 days after intervention in these patients, respectively. Because group 3 consisted of three very different cases, this group was not included in the comparative analysis between the groups.

The demographic characteristics and clinical parameters of the MG cases are summarized in Table 1; a comparison of data between groups 1 and 2 is also presented. IOL refractive power was significantly higher in cases after cataract surgery compared with cases after trabeculectomy, and a cut-off value of 25 D, with a sensitivity of 89% and specificity of 64%, was determined.

IOP in the affected eye before intervention, which provoked MG, was significantly higher compared with IOP in the other eye: in the cataract group 18.95 mmHg (14.0–20.6) vs. 12.2 mmHg (12.2–12.2) (*p* = 0.028), in the trabeculectomy group, 29 mmHg (24.35–36.8) vs. 17.0 (13.1–18.95) (*p* = 0.012).

The median time from MG to PPV treatment was 8 days (IQR 3–19.5 days), and did not differ between the groups. All cases, except for three, were treated within 55 days after development of MG. Exceptional cases were treated successfully after 198, 744, and 926 days, respectively, and only the last case needed IOP-lowering drops after PPV. The possible reason for the delay in the last case may have been moderate ocular hypertension (26.6 mmHg), which developed after cataract surgery. The other two cases were treated with other measures: the first was after cataract surgery followed by trabeculectomy; the second developed after trabeculectomy and was treated with oral acetazolamide, topical atropine and, later, anterior vitrectomy before undergoing PPV.

The median follow-up time after PPV was 90 days ((IQR) 90–286 days). The shortest follow-up time was 3 months. Normotension was achieved with PPV and additional measures, if needed, in 36 (92.3%) patients and was considered to be a successful treatment of MG. Additional measures included trabeculectomy in 5 cases, bleb revision in 2, synechiotomy in 4, trans-scleral cyclophotocoagulation in 1 case of neovascular glaucoma that involved retinal venous thrombosis before MG, and topical glaucoma treatment. IOP-lowering drops were needed in 24 (61.5%) cases; no difference between the groups was observed. The need for IOP-lowering drops before MG and after PPV decreased in the entire study group, and in all groups separately. The distribution of patients by the number of IOP-lowering medications used before MG and after MG was treated with PPV is shown in Table 2. A similar effect was observed within the groups. Successful cases were divided into groups according to the need for topical IOP-lowering treatment after PPV: drop-free group (15 cases (41.7%)) and drop-needed group (21 cases (58.3%)). The former group had significantly more drop-free patients before the development of MG compared with the drop-needed group: 5 cases (33.3%) and 1 case (4.8%), respectively (*p* = 0.023). No other parameter was statistically different between these groups.

Deepening of the AC was achieved in 90.3% of cases; restoration of normal or deep AC was achieved in 75% of cases after PPV. A significant difference was observed within the first two groups: 80% of cataract and 33.3% of trabeculectomy cases exhibited normal or deep AC after treatment with PPV (*p* < 0.05). No association between AC status and IOP after PPV was observed.

Three (7.7%) MG cases were considered to be unsuccessful due to failure to reach normal IOP with PPV and additional measures. One case was after a trabeculectomy, which was treated with atropine afterward for several years. Discontinuation of atropine drops always led to AC flattening and IOP elevation. After performing PPV, the AC remained flat, with IOP >30 mmHg. A trabeculectomy and later revisions of bleb were also unsuccessful. The other 2 unsuccessfully treated cases of MG developed 1 and 4 days after cataract surgeries with implantation of 35 D and 25 D IOLs, both underwent PPV 8 days after MG diagnoses, previously treated with 3 and 2 IOP-lowering medications, respectively, for angle-closure glaucoma and maximal IOP-lowering therapy during MG. Ocular hypertension (22.0 and 32.0 mmHg) persisted despite additional measures (AC restoration to normal depth, maximal topical treatment and, later, trabeculectomy, trans-scleral cyclophotocoagulation).

Complications of varying severity after MG and PPV were observed in 11 cases: choroidal detachments with spontaneous resolution (*n* = 2); retinal detachment (*n* = 1); constant mydriasis (*n* = 1) in the cataract group and neovascular glaucoma (*n* = 1, which had experienced retinal venous thrombosis before the event); obstruction of filtrating zone by iris (*n* = 1) and by blood clot (*n* = 1); posterior synechia formation causing IOP rise (*n* =4 (all resolved after surgery)) in the trabeculectomy group. The cataract group experienced significantly fewer complications than the trabeculectomy group (17.4% vs. 53.8%, respectively).

## 4. Discussion

We report 39 cases of MG, of which 23 developed after cataract surgery. According to our literature search, this is the largest number of MG cases after this type of surgery to be published to date [5,6]. All cases were treated with PPV. Although some centers follow stepwise treatment for MG [2,4,5], we use a more definitive approach. Because PPV addresses the main ocular structure involved in the pathogenesis of MG, we tend to perform PPV as first-line treatment by applying other measures (cycloplegics, topical and systemic IOP-lowering medications) as adjunctive therapy while waiting for surgery.

While evaluating risk factors for MG, we observed a typical tendency in our study group. Our patients were mainly hyperopic females with short eyes and high refractive power corneas treated for glaucoma. Although differences in the prevalence of hyperopia and mean axial length between the cataract and trabeculectomy groups were not statistically significant, there was a tendency toward shorter eyes and more hyperopic patients in the MG group after cataract surgery. This observation was supported by the derivative parameter―IOL refractive power. MG cases after cataract surgery had been implanted with IOLs of significantly higher diopters with a cut-off value of 25.0 D. This difference may reflect different pathological mechanisms involved in the development of MG after cataract surgery and trabeculectomy.

An additional interesting and new detail is that MG that developed after cataract surgery was associated with IOP. Naturally, the trabeculectomy group had a higher IOP and a larger difference between the eyes before the surgery than did the cataract group. However, eyes before cataract surgery, which later provoked MG, also had a significantly higher IOP than their contralateral eyes. Therefore, we hypothesize that in addition to ocular hypertension (a known risk factor for MG [8]), a slightly higher IOP compared with the other eye could be a risk factor and should be considered while planning cataract surgery, especially in anatomically predisposed eyes. Whether preoperative lowering of IOP in such eyes could minimize the risk of MG, however, is unclear.

Our study confirms the high effectiveness and acceptable safety profile of complete PPV with posterior capsulotomy, iridectomy and, in phakic cases, phacoemulsification with IOL implantation for treating MG. We observed only one vision-threatening complication (retinal detachment) that could be directly linked to PPV. Additionally, we observed no recurrence of MG. Except for some examples with obvious treatment failure before PPV (e.g., cycloplegics and anterior vitrectomy), our study was not designed to compare PPV with other treatment modalities.

According to the literature, the rate of immediate success with medical treatment is low and recurrence is high if cycloplegics are withdrawn [8]. It is difficult to discuss laser treatment modalities, such as laser capsulotomy, hyaloidotomy, and cyclophotocoagulation, confidently because the study groups were extremely small [8]. A study involving >10 cases reported an initial success rate of 67% after Nd:YAG laser hyaloidotomy, which diminished to 46.7% after 3 months [9]. Surgical options, described in the most recent review, are vitreous aspiration (we regard this to be a dangerous approach), anterior vitrectomy, and PPV with or without lensectomy. Although initial success is comparable between PPV and anterior vitrectomy, as well as between PPV with and without lensectomy, the recurrence rate always favors PPV with lensectomy [8].

The largest and the most recent study of MG cases treated with PPV reported a 100% success rate and 5.2% recurrence rate with PPV [4]. As the authors noted, most recurrences were caused by fibrin reaction that blocked iridectomy and, as such, we argue whether these cases could be considered as a recurrence of MG. In our study, we observed 4 cases of synechia formation and IOP elevation after PPV. We regard them as PPV complications because the conditions were resolved easily after synechiotomies.

We did not observe associations between successful MG treatment and the status of AC after PPV. However, there was a significant difference between MG cases after cataract surgery and a trabeculectomy. Normal AC depth was achieved in most cataract cases (80%) and in only one-third of cases after a trabeculectomy. This could also reflect the different mechanism of MG. The retrospective nature of this study precluded us from evaluating other anterior chamber structural parameters, such as iris configuration or the position of the ciliary body, among others.

Apart from successful treatment of MG, we demonstrate the possibility for glaucoma patients to reduce the need for IOP-lowering drops after PPV. Awareness of this should lower the threshold for performing simultaneous PPV, especially in the era of 23 G and higher PPV during other intraocular procedures in glaucomatous and anatomically predisposed eyes. Such a strategy would not only be effective in MG prevention, but also as a measure to withdraw or, at least, decrease topical treatment.

## 5. Conclusions

MG after cataract surgery and trabeculectomy shared typical risk factors, including female sex, angle-closure glaucoma, and hyperopia. In addition, cases after cataract surgery tend to be implanted with IOLs of higher refractive power compared with trabeculectomy cases and to have a slightly higher IOP in the affected eye compared with the other eye, which could be also be regarded as indicating a higher risk of MG. Whether this risk could be minimized by preoperative IOP lowering remains unclear.

PPV offers reliable and immediate treatment of MG with a low complication rate, as well as the possibility for glaucoma patients to reduce or discontinue IOP-lowering medications. Therefore, simultaneous PPV in anatomically predisposed eyes with glaucoma undergoing other intraocular surgery may be beneficial and less risky in the long-term.

## Figures and Tables

**Table 1:** Clinical characteristics of patients with malignant glaucoma (MG).

Clinical Characteristics	Total (*n* = 39 Cases)	Cases after Cataract Surgery (*n* = 23)	Cases after Trabeculectomy (*n* = 13)	*p*
Female sex (%)	87.2	91.3	92.3	0.917
Age (years)	69.6 ± 11.5	57.3 ± 13.2	70.9 ± 15.3	0.500
Glaucoma (%)	84.6	73.9	100	0.052
Angle-closure glaucoma (%)	66.7	60.8	76.9	0.332
MG in other eye (%)	15.4	21.7	7.7	0.271
Hyperopia (%)	48.6	60.0	41.7	0.298
Advanced cataract (%)	15.4	17.4	15.4	0.878
Lens thickness (mm)	5.0 (4.4–5.2)	5.1 (4.4–5.2)	5.01 (3.2–5.1)	0.204
IOL refractive power (D)	24.5 (23.75–27.0)	25.5 (25.0–29.0)	24.0 (22.5–24.0)	0.010
25 D or higher IOL (%)	36	61.5	10.0	0.005
AC depth (mm)	2.32 (2.06–2.3)	2.34 (2.04–2.42)	2.26 (2.2–2.32)	0.556
Axial length (mm)	21.49 ± 1.1	21.18 ± 1.2	22.0 ± 0.6	0.158
Corneal power (D)	44.88 (44.000–46.375)	44.19 (43.437–46.813)	45.38 (44.875–45.750)	0.328
Astigmatism (D)	0.5 (0.38–0.88)	0.625 (0.5–1.06)	0.5 (0.25–1.0)	0.485
Intraocular pressure (mmHg)				
during MG episode	40 (31.6–41.4)	37 (29.0–40.05)	41 (39.0–41.4)	0.209
in affected eye before MG	21 (16.2–26.3)	19 (14.0–20.6)	29 (24.4–36.8)	0.006
difference between the eyes before MG	8 (1.6–15.45)	5 (1.2–8.4)	16 (10.9–17.9)	0.041
Time to MG development (days)				
Median (interquartile range)	3.5 (1–11)	4.5 (1–11)	2 (1–6)	0.390
longest time	34	34	18	

Normally distributed data were compared using *t*-test and presented as mean ± standard deviation. Non-normally distributed data were compared using nonparametric Mann-Whitney *U* test and presented as median with interquartile range. Anterior chamber; IOL, intraocular lens; D, diopters.

**Table 2:** Distribution of patients by the number of intraocular pressure-lowering medications used before development of malignant glaucoma (MG) and after it was treated with pars plana vitrectomy.

Number of IOP Lowering Medications	Before Surgery and Malignant Glaucoma, *n* (%)	After Treatment of MG with Pars Plana Vitrectomy, *n* (%)	*p*
0	6 (15.4)	15 (38.5)	0.012
1	7 (17.9)	4 (10.3)	0.328
2	15 (38.5)	7 (17.9)	0.047
3	7 (17.9)	8 (20.5)	0.772
4	4 (10.3)	5 (12.8)	0.731

IOP, intraocular pressure; NPar McNemar Test was applied.
